# Anti-oxidation enhancement, inflammation alleviation, and microbial composition optimization of using tussah (*Antheraea pernyi*) silk fibroin peptides for hyperglycaemia remission

**DOI:** 10.1371/journal.pone.0317891

**Published:** 2025-01-23

**Authors:** Rui Mi, Xuejun Li, Yajie Li, Xingfan Du

**Affiliations:** Liaoning Ocean and Fisheries Science Research Institute, Liaoning Academy of Agricultural Sciences, Dalian, PR China; Alexandria University, EGYPT

## Abstract

**Objective:**

This study aimed to evaluate the positive effects on anti-oxidation, anti-inflammation, and microbial composition optimization of diabetic mice using tussah (*Antheraea pernyi*) silk fibroin peptides (TSFP), providing the theoretical foundation for making the use of silk resources of *A*. *pernyi* and incorporating as a supplement into the hypoglycemic foods.

**Method:**

The animal model of diabetes was established successfully. Alloxan-induced diabetic mice were orally administered using TSFP, and the hypoglycaemic effects in vivo were systematically investigated.

**Results:**

The results indicated that TSFP could significantly reduce the fasting blood glucose (FBG) levels and suppress the mRNA expression of glycometabolism genes of diabetic mice. In addition, the TSFP could ameliorate the lipid dysbolism and contribute to a higher anti-oxidation capacity. Moreover, TSFP could alleviate pathological damages and hinder inflammatory processes of diabetic mice. Besides, the supplementation of TSFP presented a greater ability to shape and optimize the gut microbial composition by enriching the profitable bacteria and inhibiting the pathogenic microorganisms. Correlation analysis also revealed that the abundances of functional bacteria in the TSFP-treated groups exhibited better correlations with serum parameters, which would be of positive significance for blood glucose regulation and inflammation remission.

**Conclusions:**

These results collectively corroborated the feasibility and superiority of using TSFP for hyperglycaemia remission via anti-oxidation enhancement, inflammation alleviation, and microbial composition optimization, contributing to a safely feasible and biologically efficient strategy for improving anti-diabetic effects.

## Introduction

Diabetes mellitus (DM) is regarded as a serious health issue. Over the past 30 years, its prevalence has been steadily on the rise. Currently, it affects more than 500 million people globally, and this number is expected to keep increasing in the coming period [1]. DM is a metabolic disease caused by insufficient insulin, and insulin action malfunctions. DM causes disturbances in the metabolism of carbohydrates, fats and proteins characterized by elevated fasting and postprandial blood sugar levels [2, 3]. According to insulin dependence, diabetes is divided into insulin dependent diabetes mellitus (IDDM) Type 1 diabetes mellitus (T1DM) and non-insulin dependent diabetes mellitus (NIDDM) Type 2 diabetes mellitus (T2DM) [4, 5]. T1DM is mainly caused by genetic factors [6], and T2DM is characterized by pancreatic beta cell dysfunction, dedifferentiation, and death [7].

Conventionally, insulin and various hypoglycemic drugs are available for the treatment of DM [8]. However, long-term utilization of insulin or synthetic agents will result in certain adverse effects on the organs, such as drug-resistant generation, inactivation of the beta cell specific transcription factors, metabolic burden exacerbation, and heart failure occurrence [9]. Therefore, the search for safer and more natural hypoglycemic agents is on the rise. Some natural substances serve as potential sources of hypoglycemic supplements and are widely employed in the prevention of diabetes. Numerous animal and plant sources have been reported to exert beneficial impacts on combating diabetes due to their rich chemical diversity, such as polysaccharides and polyphenols [10, 11]. Among them, bioactive peptides extracted from proteolytic substances have been studied as potential nutraceuticals, providing novel therapeutic applications for the prevention or control of diabetes [12].

Tussah silkworm (*Antheraea pernyi*) is an economic insect species mainly reared in the northern part of China. Its output accounts for about 90% of the world’s total output for silk production and consumed food [13]. Tussah silk is a type of natural fiber that contains two major proteins, namely silk sericin and silk fibroin. Tussah silk fibroin peptides (TSFP) are the hydrolyzed products contained a variety of amino acids with high value utilization and processing of tussah silk fibroin, such as alanine, glycine, tyrosine, serine, aspartic acid and arginine [14, 15]. Previous studies have shown that TSFP possessed many medicinal properties and biological effects in exercising mice, including immobilizing enzymes, forming biomaterials, preventing DNA damage, as well as enhancing insulin sensitivity and glucose uptake [16, 17]. However, there are relatively few studies on the comprehensive effects of TSFP on anti-oxidation, anti-inflammation, and microbial composition optimization for alleviating hyperglycemia.

Thus, this study was conducted to evaluate the positive effects on anti-oxidation, anti-inflammation, and microbial composition optimization of diabetic mice using TSFP. The research would provide innovative insights into the functional application of *A*. *pernyi*, thereby contributing to a safely feasible and biologically efficient approach for alleviating the biochemical imbalance, oxidative damage, inflammatory lesion, and gut microbiota dysbiosis caused by hyperglycemia.

## Materials and methods

### Preparation of TSFP

With the method mentioned in the references [13], TSFP samples were obtained through the process of dissolving the degumming tussah silk at low temperature, hydrolysis temperature control, desalting and spray drying. The distribution of molecular weight range of TSFP was less than 5,000 Daltons which detected by gel chromatograph (ELEOS System Wyatt).

### Animal experiments

The Kunming (KM) mice (25–30 g body weight, BW) were obtained from the Experimental Animal Centre of Dalian Medical University (Dalian, China), and the production licence number was SCXK (Liao) 2018–0007. Individuals were fed on a basic diet and water under standard environmental conditions for one week [18]. After acclimatization period, the animals were intraperitoneally injected with alloxan monohydrate (ALX, Sigma-Aldrich Fine Chemicals, St. Louis, MO, USA) at a dose of 200 mg/kg·BW after an 18-h fasting [19]. One week later, blood samples were collected from the tails and blood glucose levels were evaluated. The animals with fasting blood glucose (FBG) levels between 11 and 30 mmol/L were further used for the experiments as the diabetic model [20]. Then, the individuals were equally split into three groups (10 individuals for each treatment). The model group (M group) was treated by oral gavage of distilled water daily. The other groups were treated with TSFP at doses of 2 (low dose group, L group) and 6 g/kg·BW (high dose group, H group) by daily oral gavage, which were designed in accordance with the previous study and modified from the preliminary experimental results [14]. This study was conducted according to the rules and regulations of the Experimental Animal Ethics Committee of Dalian Medical University (Dalian, China) during the entire experimental period (Ethical approval number: AEE20001). The appropriate anesthetic agent (ketamine, 100 mg/kg·BW) was employed and the procedures were reviewed and updated based on the latest scientific knowledge to further enhance the well-being of the animals involved. All animal procedures were performed in accordance with strict ethical guidelines and the carbon dioxide method was used for euthanasia to minimize distress and ensure a quick and painless end.

### BW and FBG determination

After administration of TSFP, the BW and FBG levels were measured at days 0, 7, 14, and 21. Blood samples were collected from the orbit after fasting overnight and the FBG levels were determined by a glucometer.

### Biochemical parameters measurement in serum

All treated animals were fed for 3 weeks. After fasting for 12 h, blood samples were collected for biochemical parameters analysis. Serum insulin (INS) was measured by a double-antibody method using a rat insulin kit (Nanjing Shenbeijia Biotechnology LTD, Nanjing, China). Glycosylated serum protein (GSP), triglyceride (TG), total cholesterol (TC), low-density lipoprotein cholesterol (LDLC), high-density lipoprotein cholesterol (HDLC), malondialdehyde (MDA), dismutase (SOD), catalase (CAT), nitrogen (BUN) and creatinine (CR) levels were evaluated using enzyme-linked kits purchased from Nanjing Jiancheng Bioengineering Institute.

### Histomorphological analysis of tissue

After 21 days of treatment, pancreas, liver and kidney were dissected from the individuals, washed thoroughly, and cut into pieces of 0.5×0.5×0.2 cm, and then fixed with formalin. The samples were then dehydrated and cleared, and embedded by paraffin. Slices of 4 mm thickness were collected using a leica RM2135 microtome for serial sections, HE staining and microscopic imaging.

### Preparation of cDNA and qRT-PCR

Total RNA of the liver tissues was extracted using TRIZOL kit (Invitrogen). The extracted RNAs were dissolved in RNase-free water and stored at −80°C until use. The total RNA concentration was estimated, and then the cDNA was synthesized using the SYBR PrimerScrip RT-PCR kit with 0.5 μg of total RNA.

The primers used in the present study were designed in accordance with the gene sequences of glucose-6-phosphatase (G6Pase), glycogen phosphorylase (GP), interleukin-4 (IL-4), GATA-binding protein 3 (GATA-3), T cells (T-bet), and interferon-γ (IFN-γ). All these genes were the key metabolic enzymes and immune factors [18]. Glyceraldehyde-3-phosphate dehydrogenase (GAPDH) was used as an internal control. The quantitative real-time PCRs were performed using the SYBR Premix GC ExTaq kit (TAKARA biotechnology Dalian co. LTD, China). The *q*PCR was performed with ABI7300 (Applied Biosystem, USA). The *q*PCR data of relative gene expression were rectified with the 2^-△△Ct^ method of quantitative analysis.

### Gut microbiota analysis

The total DNA was extracted from feces of individuals using a DNA Extraction Kit at the beginning and the end of the trial. Agarose gel electrophoresis was used to estimate the completeness and concentration of DNA. The V3-V4 regions of 16S rRNA genes were amplified and sequenced on Illumina MiSeq platform. The amplicon libraries were generated, and then the paired-end reads obtained through sequencing were processed and joined. Afterward, the reads were assembled, denoised, and merged by DADA2 plug-in unit. The ASVs were attributed to the taxonomy on the basis of Greengenes database, then the ASV abundance tables were established. Singletons were discarded to enhance data analysis efficiency, and the ASV abundance tables were standardized by the reads on basis of the least reads.

### Statistical analysis

Alpha diversity indices were determined by PAST v3.0. Boxplots were drawn to display the richness and diversity of the samples by R v3.6.1 platform. The relative abundances of predominant bacteria were visualized with the “ggplot2” package in the R platform. Spearman’s correlation was analyzed to evaluate possible associations between the serum parameters and gut microbial composition using “heatmap” package. Duncan’s multiple range tests were conducted to assess the mean differences. The data analysis was conducted by SPSS software v. 21.0, and the levels of significance were expressed as *P* < 0.05.

## Results

### Effects of TSFP on BW, FBG, INS and GSP levels in diabetic mice

**Fig 1:**
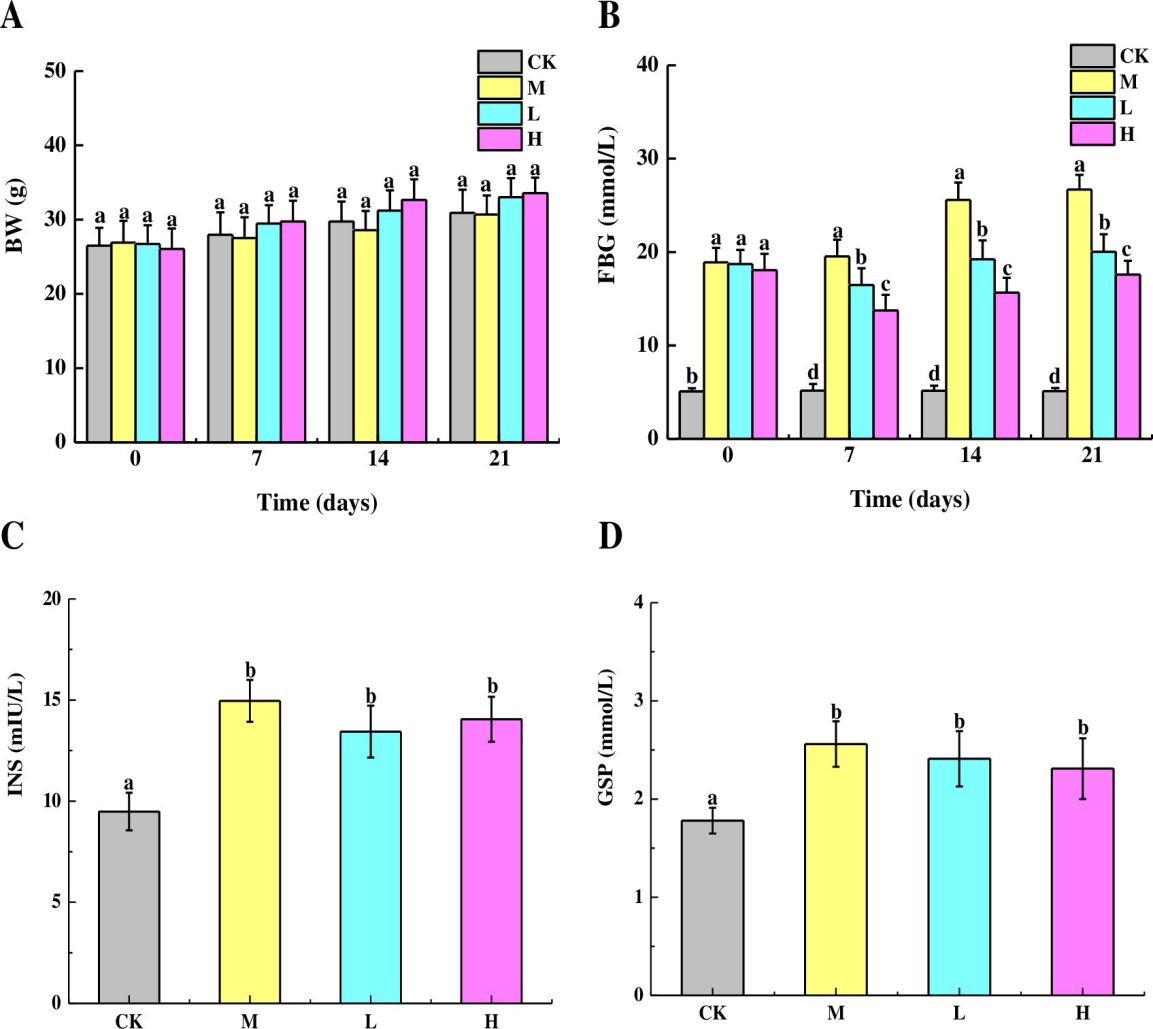
Effects of TSFP on the BW (A), FBG (B), INS (C), and GSP (D) levels of diabetic mice. Values are means and standard errors of three replicates. Bars marking different letters are significantly different (*P* < 0.05). The capital letters CK, M, L and H represent the control group, model group, low dose group and high dose group.

The BW changes with groups were shown in Fig 1A. During the feeding trial, the BW of the animals in all the groups increased steadily, while no obvious differences were observed among all the treatments. Even so, the values of BW presented by mice in the TSFP-treated groups continued to be slightly higher than that of the M group.

As shown in Fig 1B, the FBG levels of the experimental groups were significantly higher than that of the control group after injection, indicating that the diabetic mice model was established successfully. At day 7, the FBG levels of the H group decreased distinctly compared with the model group (*P* < 0.05). Thereafter, the FBG levels of the TSFP-treated groups were all lower visibly than that of the model group, with the control group showing the lowest levels (*P* < 0.05).

The effects of TSFP on the INS levels of diabetic mice were shown in Fig 1C. The serum INS levels of diabetic model group were significantly higher than the control group (*P* < 0.05). In addition, no significant differences were detected between the M and TSFP-treated groups (*P* > 0.05). The INS levels of the TSFP-treated groups lowered slightly than the M group, and the L group produced the better INS value than the H group.

As shown in Fig 1D, the GSP levels of the three experimental groups were markedly higher than the control group (*P* < 0.05). The maximum values presented in the M group, followed by the L and H groups. As before, the changes of the GSP levels did not reach significant levels among the experimental groups (*P* > 0.05).

### Effect of TSFP on blood lipid levels in diabetic mice

**Fig 2:**
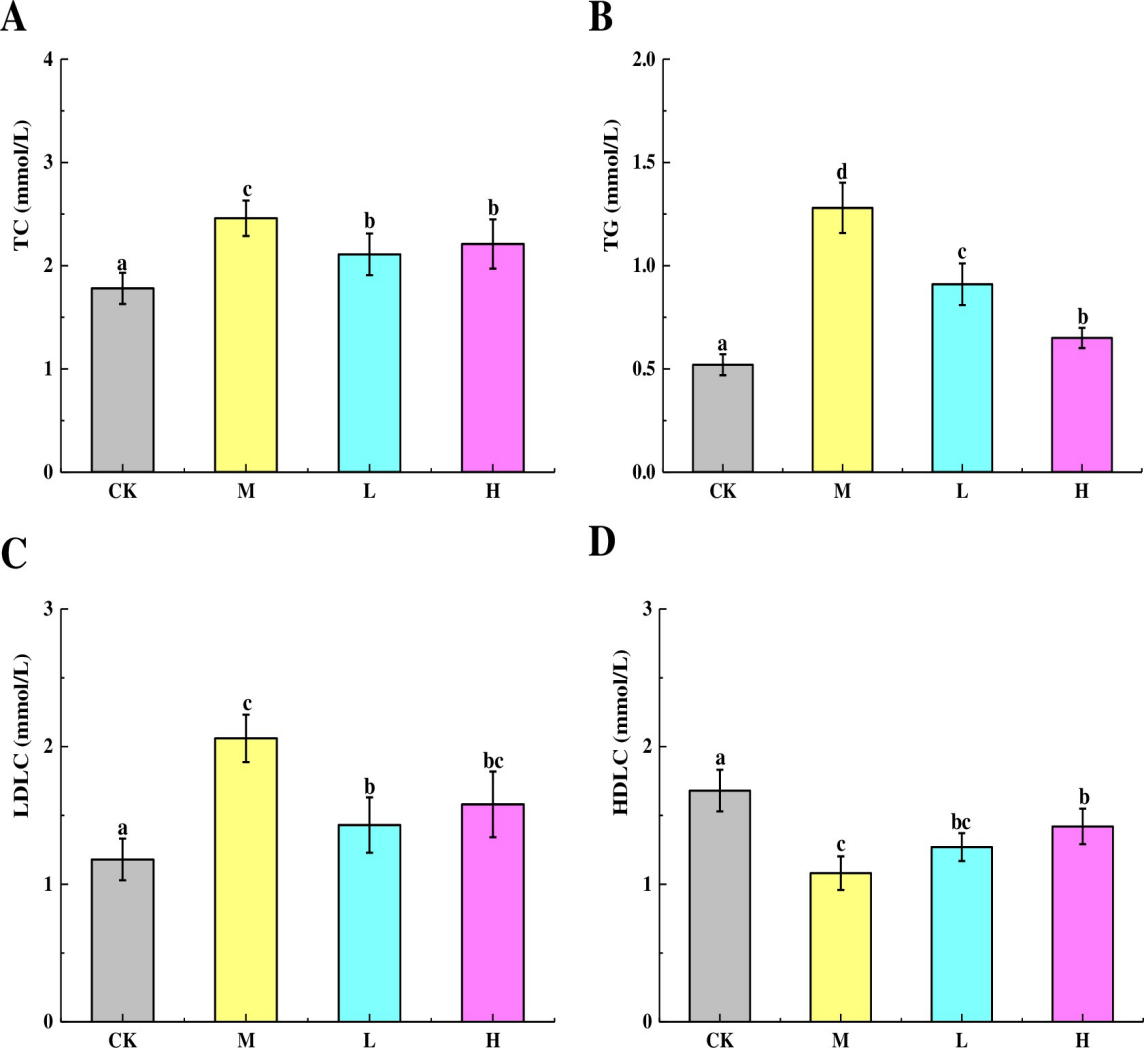
Effects of TSFP on the blood lipid levels of diabetic mice. The levels of TC (A), TG (B), LDLC (C), and HDLC (D). Values are means and standard errors of three replicates. Bars marking different letters are significantly different (*P* < 0.05). The capital letters CK, M, L and H represent the control group, model group, low dose group and high dose group.

The effects of TSFP on the blood lipid levels of diabetic mice were shown in Fig 2. The TC levels of the treatments were signally higher than the control group (*P* < 0.05), and the M group displayed higher TC values than the TSFP-treated groups (Fig 2A). As for the TG levels, the highest values were observed in the M group, followed by the L, H, and control groups (*P* < 0.05) (Fig 2B). Additionally, the highest levels of LDLC were presented in the M group, followed by the H, L, and control groups (*P* < 0.05) (Fig 2C). The lowest HDLC values appeared in the M group, while the higher HDLC values were detected in the control group, followed by the H and L groups (*P* < 0.05) (Fig 2D).

### Effects of TSFP on antioxidant activity and renal function in diabetic mice

**Fig 3:**
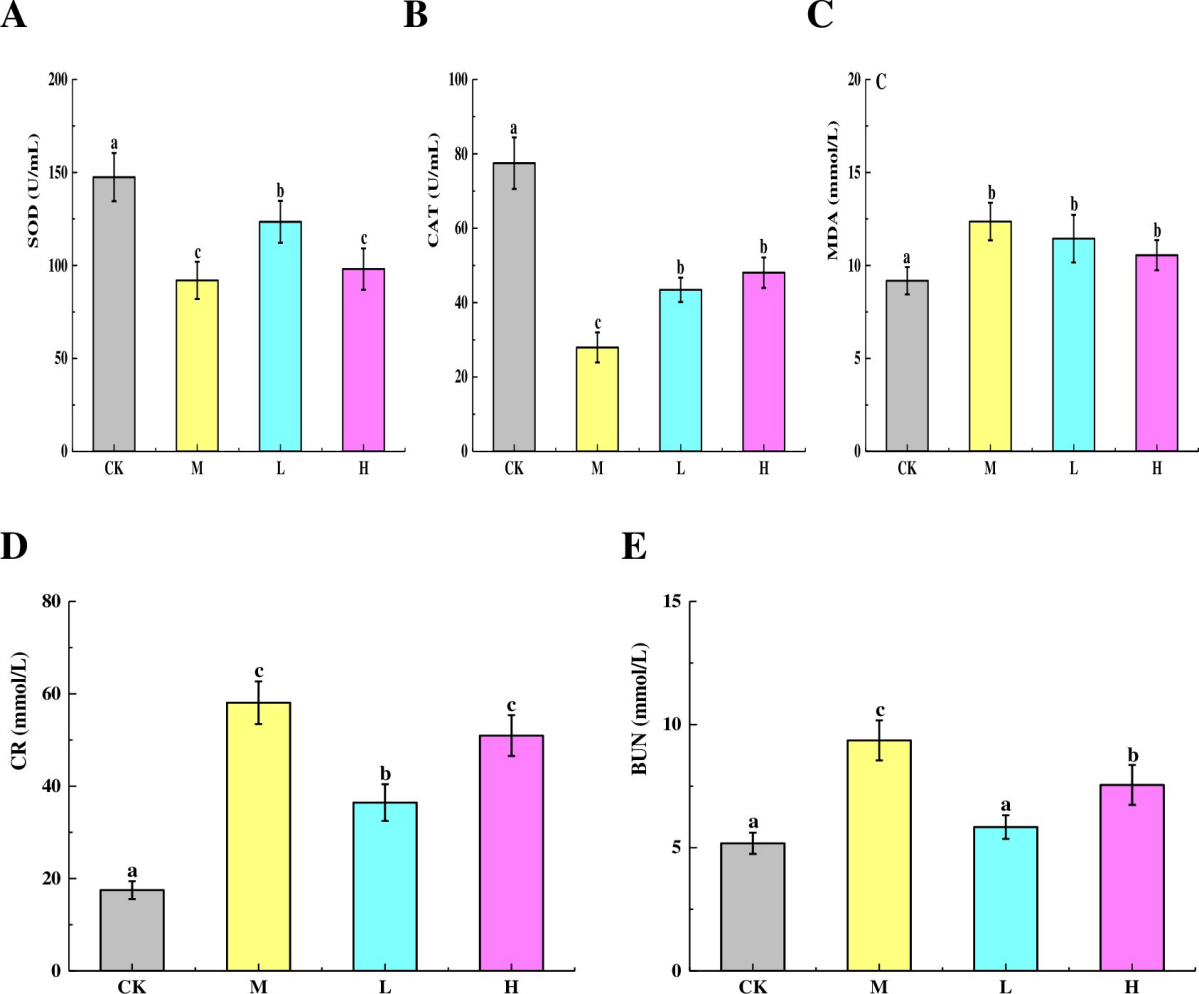
Effects of TSFP on the levels of SOD (A), CAT (B), MDA (C), CR (D) and BUN (E) of diabetic mice. Values are means and standard errors of three replicates. Bars marking different letters are significantly different (*P* < 0.05). The capital letters CK, M, L and H represent the control group, model group, low dose group and high dose group.

The effects of TSFP on the antioxidant enzyme levels of diabetic mice were shown in Fig 3. The control group exhibited an obvious higher SOD activity than others (*P* < 0.05), followed by the L group, and no distinct differences were detectable between the H and M groups (*P* > 0.05) (Fig 3A). The control group showed the highest CAT activities among treatments, while the lowest values presented in the M group (*P* < 0.05). Moreover, no obvious differences in CAT activities were observed in the TSFP-treated groups (*P* > 0.05) (Fig 3B). Differently, less variations of MDA activities were observed with groups (*P* > 0.05). Even so, the H group demonstrated marginally lower MDA activities compared to the other groups, with the M group exhibiting the highest levels (Fig 3C).

The effects of TSFP on the CR and BUN levels of diabetic mice were shown in Fig 3D and 3E. The CR and BUN values in the M group increased dramatically compared with the other groups, followed by the H and L groups (*P* < 0.05). As expected, the control group displayed the lowest CR and BUN levels. It was noted that the contents of CR displayed in the M group were a little higher than the H group, and no obvious differences in the BUN levels were detected between the L and control groups (*P* > 0.05).

### Effects of TSFP on inflammatory response in diabetic mice

**Fig 4:**
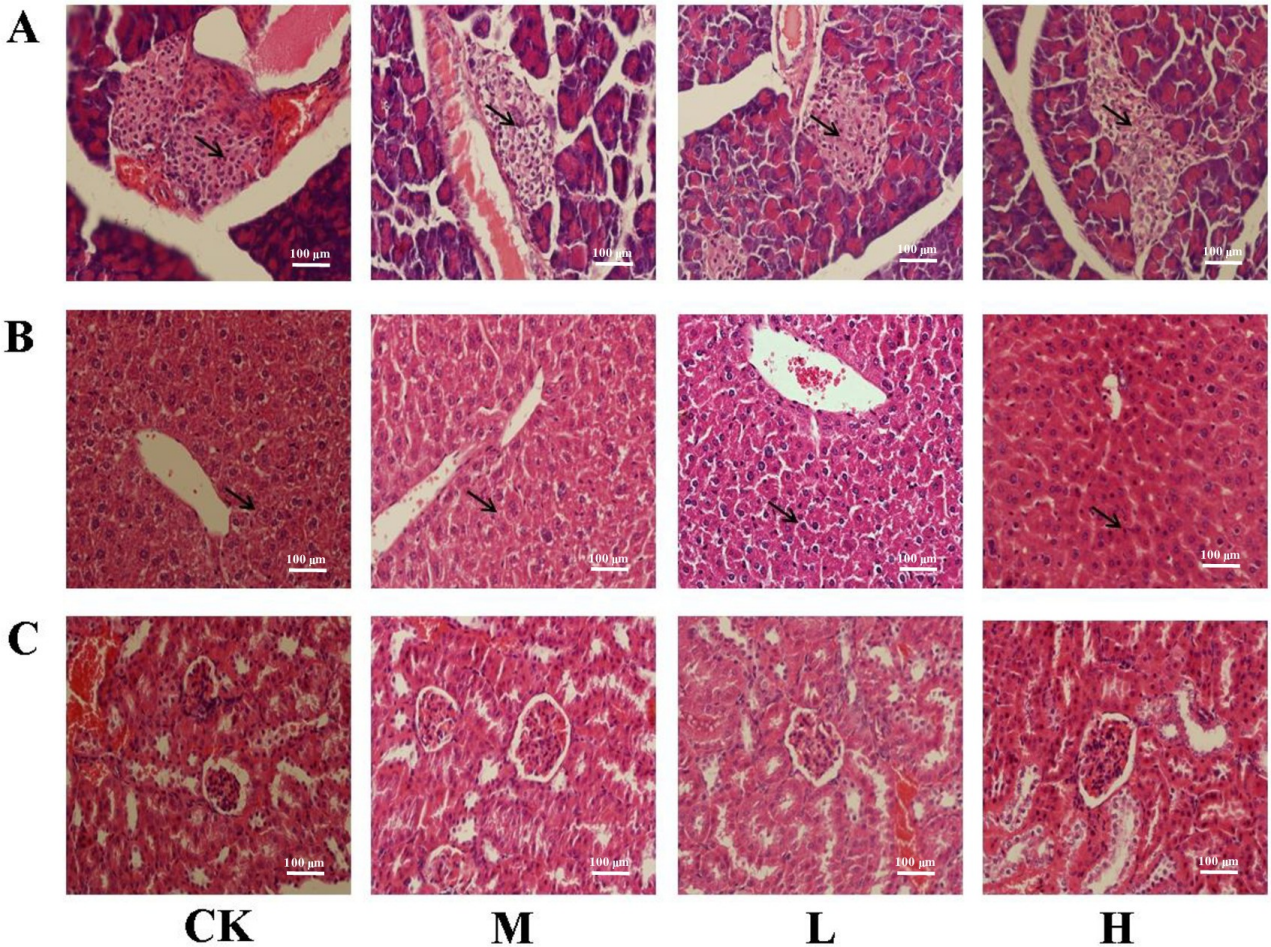
Effects of TSFP on histopathological changes of pancreas (A), liver (B), and kidney (C) of diabetic mice. HE staining was used for the analysis of histological changes. The black arrows in Fig 4A pointed to the pancreatic islet cells, and the black arrows in Fig 4B pointed to the liver cells. The capital letters CK, M, L and H represent the control group, model group, low dose group and high dose group.

Pathological analysis was conducted to study the effects of TSFP on the pathological changes of the pancreas, liver and kidney. The black arrows in Fig 4A pointed to the pancreatic islet cells. In the control group, the pancreatic islet cells were compact and had clearly visible cell nuclei. However, in the M group, cell degranulation, vacuolar degeneration, and lymphocyte infiltration were observed, indicating that the immune system and cell structure of mice were severely damaged in the pancreas. Upon administration of TSFP, less vacuolar degeneration and infiltration of inflammatory cells were observed compared to the M group, indicating that TSFP can effectively protect islet cells from hyperglycemic damage (Fig 4A).

The black arrows in Fig 4B pointed to the liver cells. Liver cells of the control group were arranged orderly around the central vein, and the entire structure was intact. There was no obvious fat vacuole in the cytoplasm, nor were there any inflammation or infiltration. In contrast, the liver cells in the M group were disordered and abnormal, and the pathological changes, including edema and focal necrosis, had occurred in some extent. The hepatic morphology of the TSFP-treated groups was improved distinctly, the cells lined up tightly, and the edema reduced observably (Fig 4B).

As for the histopathological changes of the kidney, the glomeruli cells in the control group were distributed orderly, with a complete glomerular structure. In the M group, the mesangial regions expansion, overflowing nuclei, and vacuoles degeneration were observed. Visibly, these impairments were ameliorated in the TSFP-treated groups to a certain degree (Fig 4C). The results collectively suggested that TSFP would be an effective intervention to repair organs injuries caused by diabetes.

### Effects of TSFP on mRNA expressions of rate-limiting enzymes and immune genes

**Fig 5:**
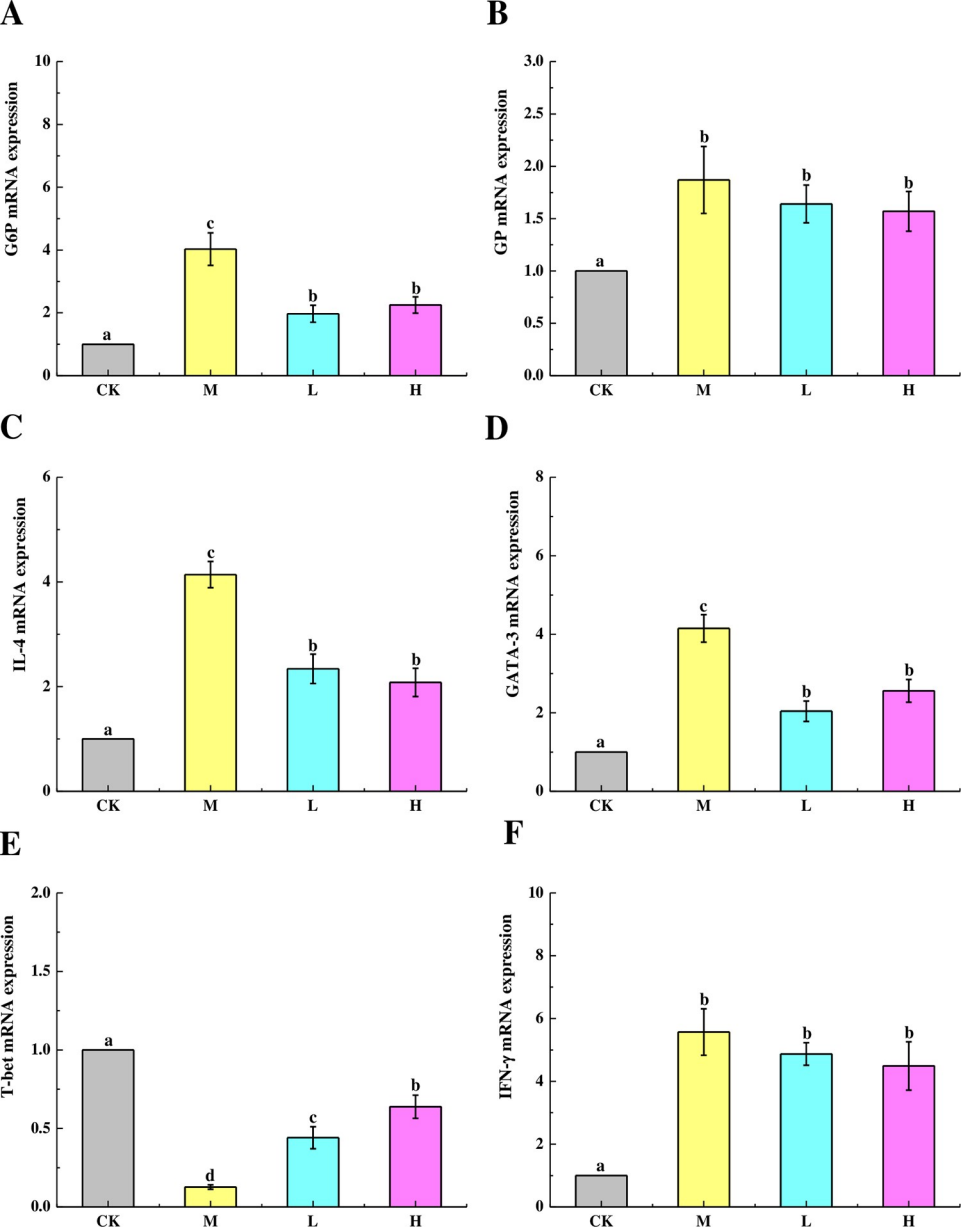
Effects of TSFP on the mRNA expression levels of G6Pase (A), GP (B), IL-4 (C), GATA-3 (D), T-bet (E), and IFN-γ (F) in the liver tissue of diabetic mice. Values are means and standard errors of three replicates. Bars marking different letters are significantly different (*P* < 0.05). The capital letters CK, M, L and H represent the control group, model group, low dose group and high dose group.

The mRNA expression levels of G6Pase and GP were assessed by a real-time quantitative PCR experiment (Fig 5A and 5B). The levels of G6Pase and GP were up-regulated significantly than the control group (*P* < 0.05), suggesting that a large number of glucose units were produced. As for the TSFP-treated groups, the G6Pase mRNA expression levels were reduced markedly compared with the M group (*P* < 0.05), whereas the TSFP did not exhibit the ability of reducing the GP mRNA expression level significantly. Even so, the GP mRNA expression levels in the TSFP-treated groups continued to be slightly lower than the M group (*P* > 0.05).

The immune factors associated with triggering diabetes, including the cytokines of IFN-γ and IL-4, the Thl/Th2 polarization key transcription factors T-bet and GATA-3 were determined and analyzed. Compared to the control group, the mRNA expression levels of the IL-4 and GATA-3 were up-regulated markedly in the M group (*P* < 0.05), indicating an apparent inflammatory reaction in the livers. In contrast, TSFP could reduce the mRNA expression levels of IL-4 and GATA-3 distinctly in the H and L groups (*P* < 0.05) (Fig 5C and 5D). Comparatively, TSFP could increase the mRNA expression levels of T-bet more obviously in the H group than the L group with the M group showing the lowest levels (*P* < 0.05) (Fig 5E). Moreover, no obvious differences in the mRNA expression levels of IFN-γ were detected among the M and TSFP-treated groups (*P* > 0.05) (Fig 5F). These results demonstrated that TSFP could exert protective effects on reducing Th cells polarization and alleviating inflammation of the diabetic mice by regulating the mRNA expression levels of immuno-related genes.

### Effects of TSFP on gut microbial composition in diabetic mice

**Fig 6:**
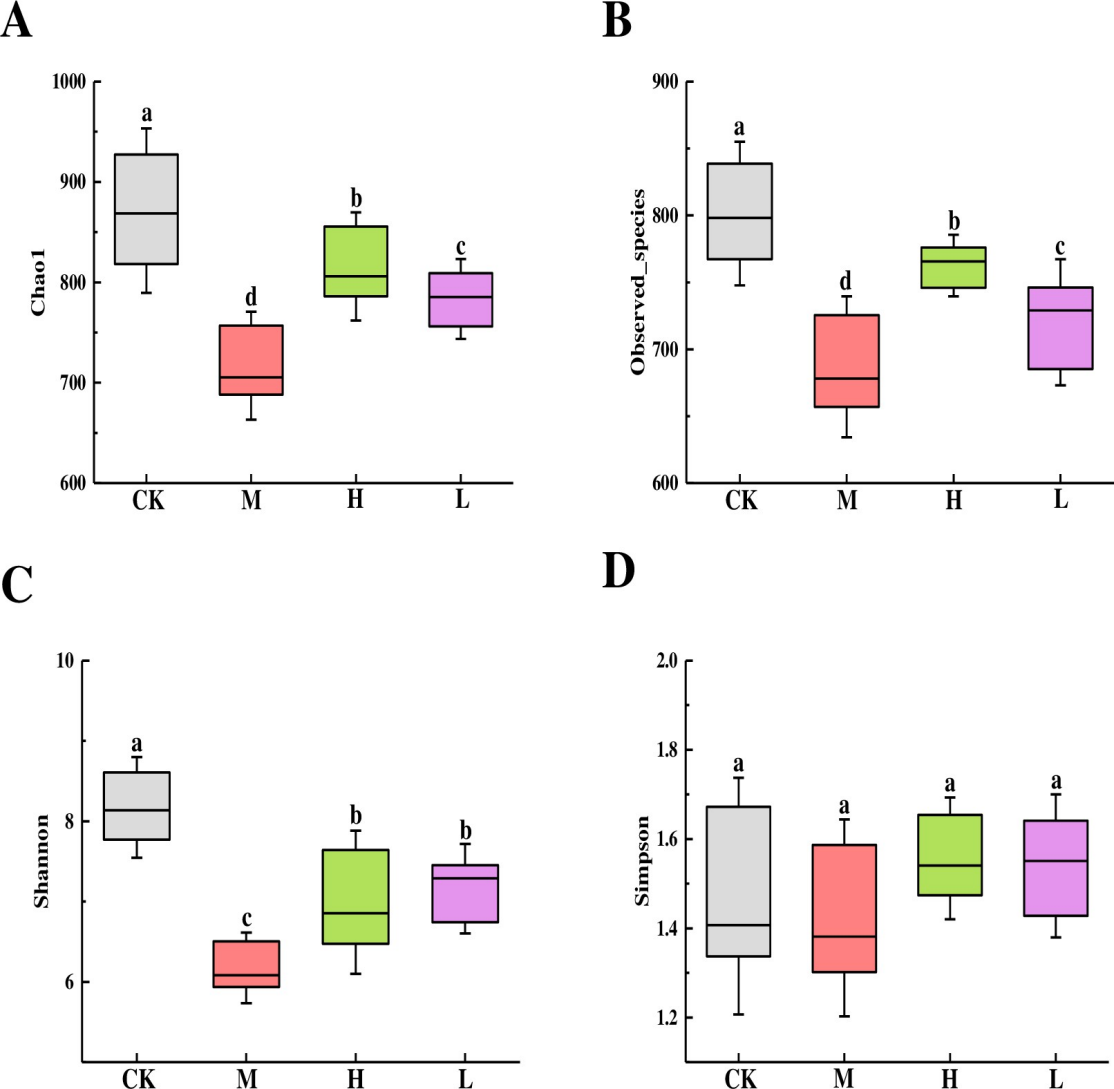
Boxplots for alpha diversity indices of Chao1 (A), Observed species (B), Shannon (C) and Simpson (D) in the diabetic mice with groups. Values are means and standard errors of three replicates. Bars marking different letters are significantly different (*P* < 0.05). The capital letters CK, M, L and H represent the control group, model group, low dose group and high dose group.

Alpha diversity indices can display the community richness and diversity with groups. As shown in Fig 6, the maximum values of Chao1 and Observed species were detected in the control group that followed by the H and L groups, and the minimums presented in the M group (*P* < 0.05) (Fig 6A and 6B). The control group displayed the highest Shannon values with the M group showing the minimums (*P* < 0.05), and no obvious differences were observed between the H and L groups (*P* > 0.05) (Fig 6C). Moreover, no apparent difference in Simpson index had occurred among the groups with the exception of the less decrease in the M group (*P* > 0.05) (Fig 6D).

**Fig 7:**
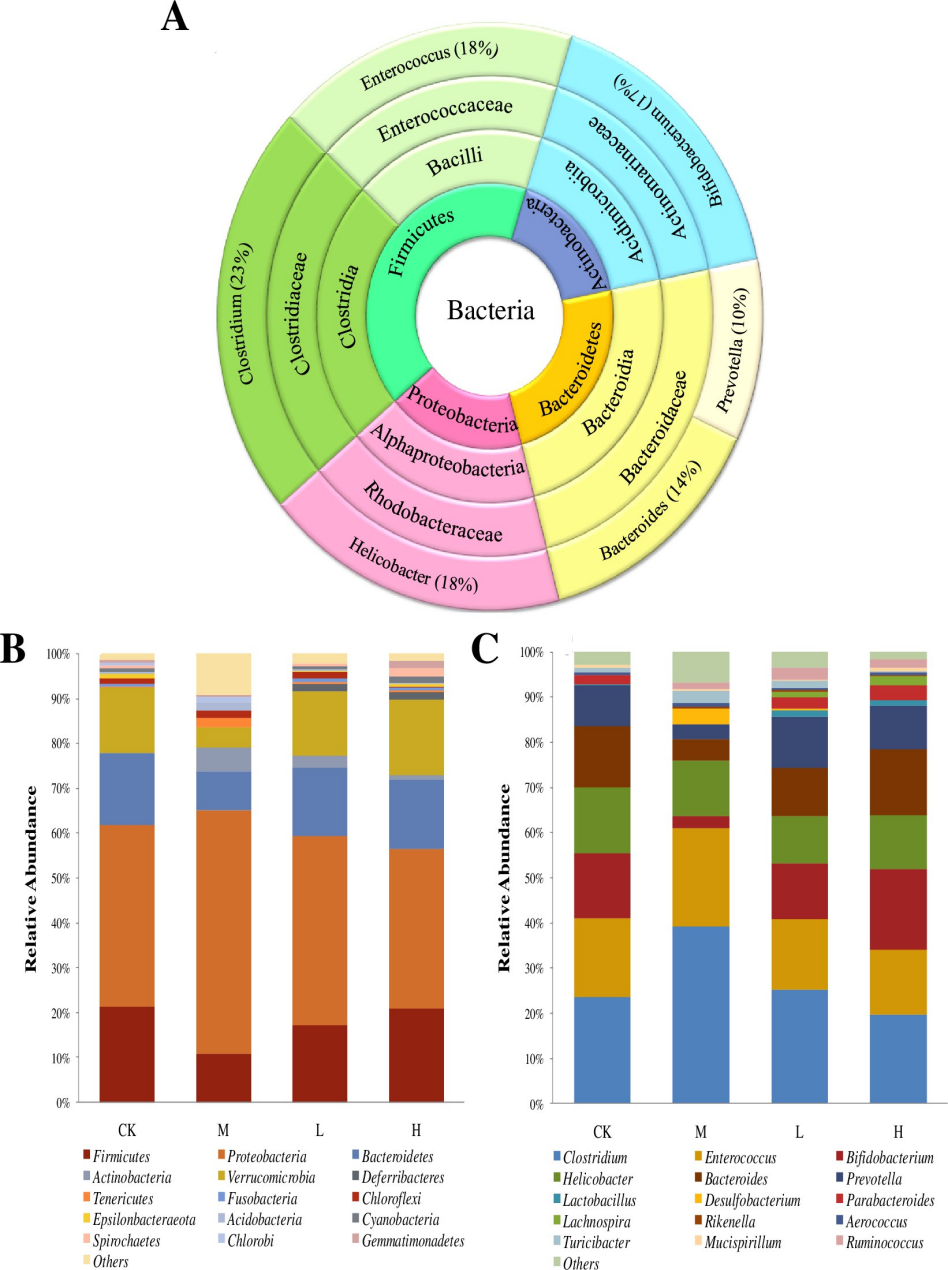
Gut microbiota composition of mice before supplementation with TSFP at four taxonomic levels (A). Effects of supplementation with TSFP on gut microbiota composition of mice at phylum (B) and genus (C) levels. The capital letters CK, M, L and H represent the control group, model group, low dose group and high dose group.

The taxonomic gut microbiota composition of mice before administration of TSFP was shown in Fig 7A. The Firmicutes, Bacteroidetes, Proteobacteria, and Actinobacteria were the predominant phyla in the samples. The dominant classes were Clostridia, Bacilli, Acidimicrobiia, Bacteroidia, Alphaproteobacteria, and the core families were Clostridiaceae, Enterococcaceae, Actinomarinaceae, Bacteroidaceae, Rhodobacteraceae, and Clostridiaceae. At genus level, higher abundances of *Helicobacter*, *Clostridium*, *Enterococcus*, and *Bifidobacterium* were detected in the samples.

Changes of gut microbiota composition of mice in the four experimental groups were shown visually, and most of the gut microorganisms in these groups were shared microbiome, while the relative abundances differed. At the phyla level (Fig 7B), the highest abundances of Bacteroidetes were detected in the control group, followed by the H and L groups, while the minimum values appeared in M group, respectively. The maximum Actinobacteria was observed in the H group that followed by the control, L, and M groups. The higher proportions of Firmicutes and Proteobacteria were detected in the M group, while no significant differences were detected in the other groups. At the genus level (Fig 7C), the higher abundances of *Bacteroides*, *Prevotella*, *Bifidobacterium* were presented in the H and L groups, and the lowest values appeared in the M group. In addition, visible increases in the abundances of *Clostridium* and *Enterococcus* were presented in the M group, whereas the lower abundances appeared in the TSFP-treated groups. Increased abundances of *Desulfobacterium* were detected in the M group, which were detected in very low abundances in the L group and even in no observed proportions in the H and control groups contrastively. Additionally, some new genera, such as *Lactobacillus*, *Parabacteroides*, and *Lachnospira*, emerged in the L and H groups to a certain extent.

### Correlation between the serum parameters and gut microbial composition in diabetic mice

**Fig 8:**
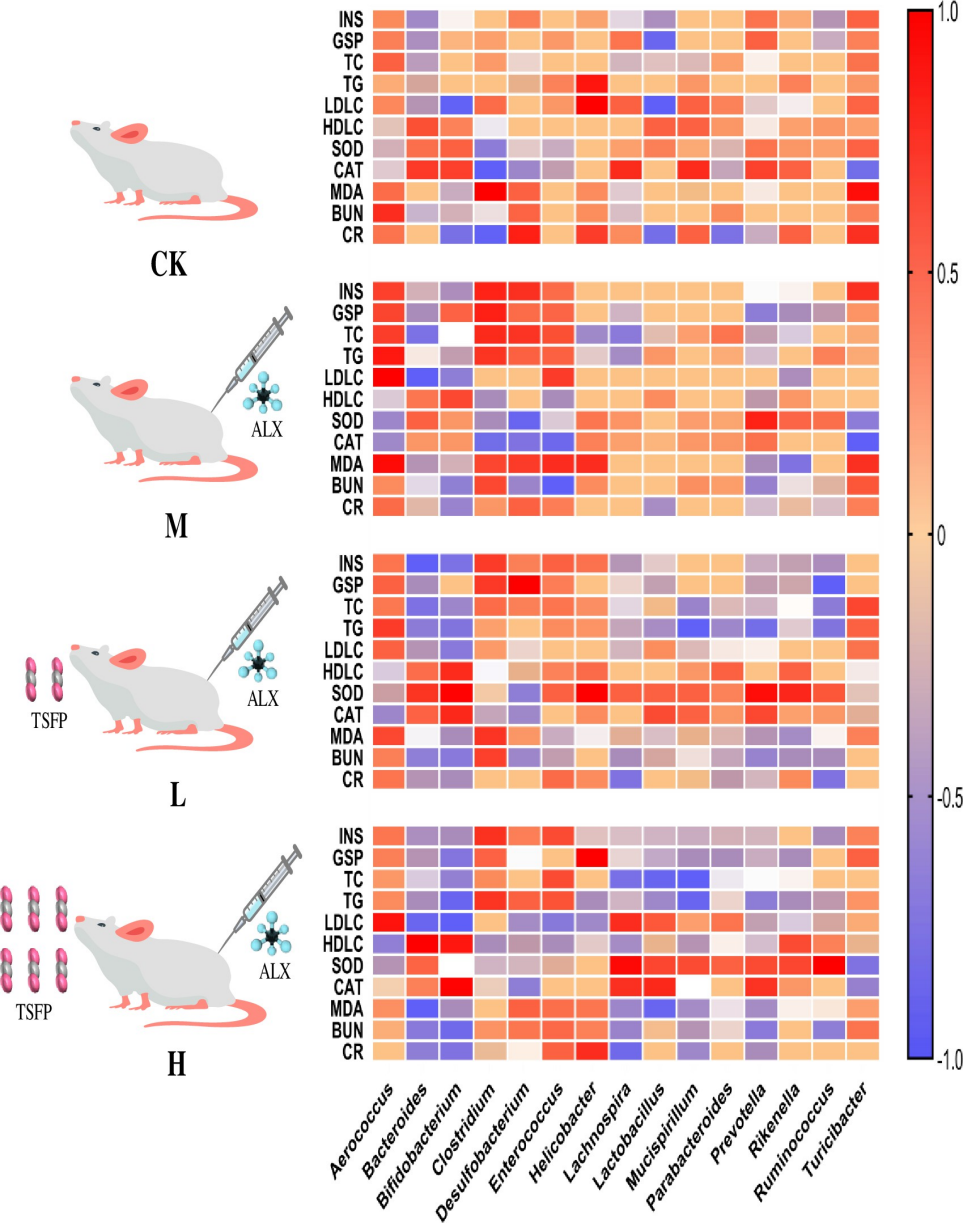
Heatmaps of Spearman’s correlations between serum parameters and gut microbial composition in diabetic mice. The colour gradient expresses the Spearman’s rank correlation coefficients between the relative abundance of each genus and each serum parameter. The capital letters CK, M, L and H represent the control group, model group, low dose group and high dose group.

To clarify the response relationships between serum parameters and gut microbial composition in diabetic mice, the correlation analysis was performed. As shown in Fig 8, the abundances of *Aerococcus*, *Clostridium*, *Desulfobacterium*, *Enterococcus*, and *Turicibacter* showed significantly positive correlation with the INS, GSP, TC, TG, MDA, BUN, and CR levels, and had a negative correlation with the HDLC, SOD, and CAT levels in the M and TSFP-treated groups. Relatively, the TSFP-treated groups exhibited lower correlations between these genera with serum parameters. In the H and L groups, the abundances of *Bacteroides*, *Bifidobacterium*, *Lachnospira*, *Lactobacillus*, and *Rikenella* were negatively correlated with the INS, GSP, TC, TG, MDA and BUN, and positively correlated with the HDLC, SOD, and CAT levels to varying degrees. In addition, the low correlations between the serum parameters and the abundances of *Lachnospira*, *Lactobacillus*, *Mucispirillum*, and *Parabacteroides* had been observed in the M group. Comparatively, the TSFP-treated groups showed more obvious correlations between functional communities and the serum parameters than the control and M groups.

## Discussion

Diabetes is a chronic metabolic disease, accompanied by oxidative stress, inflammation, and gut microbiota dysbiosis, which interact with each other and jointly play a crucial role in blood sugar control [21]. The intestinal microbiota can enhance the antioxidant capacity by producing antioxidant substances such as short-chain fatty acids and vitamins. At the same time, a healthy intestinal microbiota can maintain the integrity of the intestinal mucosal barrier and reduce the entry of endotoxins, thereby reducing the occurrence of inflammatory responses. On the contrary, an imbalance in the intestinal microbiota can lead to increased oxidative stress and intensified inflammatory responses, further affecting blood sugar control [11]. In addition, the enhancement of antioxidant capacity can reduce the damage of oxidative stress to the intestinal microbiota and maintain the balance of the intestinal microbiota. Therein, antioxidants can neutralize free radicals generated during the inflammatory process and reduce inflammatory responses. By improving antioxidant capacity, the composition and function of the intestinal microbiota can be improved, and the inflammatory level can be reduced, which is beneficial for blood sugar control. Enhancement of antioxidant capacity can also regulate inflammation-related signaling pathways and inhibit the production and release of inflammatory factors, thereby reducing the adverse effects of inflammation on blood sugar control [12]. Under an inflammatory state, the intestinal microenvironment changes, affecting the composition and distribution of the intestinal microbiota. Inflammation also consumes a large amount of antioxidant substances and reduces antioxidant capacity. At the same time, inflammatory factors can inhibit the activity of antioxidant enzymes, further weakening the function of the antioxidant system. Therefore, controlling inflammatory responses is extremely important for maintaining the balance of the intestinal microbiota and improving antioxidant capacity, and consequently is beneficial for blood sugar control [20]. In summary, the intestinal microbiota, antioxidant capacity, and inflammatory response are interrelated and jointly affect the control of blood sugar. In this study, alloxan-induced diabetic mice were administered TSFP orally, and the ameliorative effects on antioxidation, anti-inflammation, and optimization of microbial composition in vivo were systematically investigated. The changes of the FBG levels of the experimental groups indicated that the diabetic mice model was established successfully and the blood glucose would be regulated effectively by TSFP. However, the changes in BW, INS, and GSP levels did not attain a significant degree among the experimental groups in this study. This could potentially be accounted for by factors such as feeding and management conditions, individual variations, and administration levels [22, 23]. It was noteworthy that such results could also be brought about by short-term TSFP treatment. The reason might lie in the fact that the established diabetic mice model was based on a fundamental characteristic of type 1 diabetes with a short processing duration. Given that BW, INS, and GSP levels were manifestations of the overall blood sugar control over a certain period of time, it was difficult to achieve a significant impact in a short span [6]. Although not being significant, the values of BW, INS, and GSP displayed in the TSFP-treated groups remained slightly better than that of the M group, indicating that TSFP could exert the hypoglycemic effects and improve glucose metabolism in vivo effectively.

In diabetic mice, oxidative free radicals may increase dramatically and further lead to oxidative stress and tissue damage [24, 25]. The host needs TC and TG for energy accumulation, fatty acid synthesis, and cell membranes construction, while high levels of which can cause serious health problems associated with lipid peroxidation and lipid metabolism disorder [26]. Similarly, LDLC is a form of lipoprotein particle involved in cardiovascular diseases. HDLC can improve cholesterol metabolism, which would prevent atherosclerosis [27]. In this study, the highest TC, TG, and LDLC levels were presented in the M group, and the lowest values appeared in the control group. Additionally, the M group exhibited the highest levels of LDLC, with the H, L, and control groups following in succession. The results demonstrated that TSFP was capable of enhancing the regulation of lipid metabolism in diabetic mice. This might be attributed to the fact that TSFP contained large amounts of glycine and serine. Through decomposition, adsorption, and gelatinization, these substances could effectively alleviate dyslipidemia and counteract lipid peroxidation [24, 28]. Besides, the functional oligopeptides with α-glucosidase inhibitive activity presented in the TSFP would also suppress lipid accumulation and create the synergistic effects with the amino acids for improving the effectiveness of lipid metabolism and inhibiting lipid peroxidation [13]. The results were similar to the previous report, which clearly demonstrated therein that the addition of a combination of red ginseng powder and silk peptide could notably reduce hypercholesterolemia and atherosclerosis in rabbits, presumably by regulating lipid metabolism and eliminating free radicals [29].

The enzymes of SOD and CAT played a role of antioxidants by eliminating superoxide and hydrogen peroxide, and they were not consumed by the reaction [30, 31]. MDA is a genotoxic byproduct of lipid peroxidationm, which can change membrane properties and cause cross-linking polymerization of macromolecules [32]. In this study, the TSFP-treated groups displayed higher activities of SOD and CAT than the M group, while the TSFP-treated groups showed slightly lower MDA levels than the M group, implying that the SOD and CAT were more sensitive parameters than the MDA for diabetic mice in response to the TSFP treatment [26]. The results were similar to previous report, which confirmed that the fibroin peptides could prevent DNA damage, and would exert the antioxidant action of inhibiting the activity of tyrosinase [16]. These results collectively indicated that TSFP could effectively scavenge free radicals and reduce the oxidative damage in diabetic mice distinctly, which were consistent with the histopathological changes of organ tissues, where the treatment of TSFP showed fewer inflammatory reactions.

To further evaluate renal function, CR and BUN levels were measured in this study. Higher CR and BUN values were observed in the M group, and the L group showed the lowest values, followed by the H group. This indicated that the tissue structures were damaged, leading to the impairment and compromise of the renal filtration function [33], which were consistent with the results of histology observations. After TSFP administration, the levels of CR and BUN were reduced to some extent, while the L group could exert positive benefits more effectively with a certain dose-dependent manner. The results could be rationalized by the fact that the amino acids and functional groups in TSFP could improve erythrocyte immune adherence function, reduce toxins accumulation, alleviate renal fibrosis, and delay renal injury in diabetic mice, whereas the TSFP would be excessive accumulation in the H group, and might not offer equivalent contribution to renoprotective effects with lower solubility, gelation and biocompatibility [13, 24].

The liver is a pivotal organ for glucose storage and energy metabolism. G6Pase and GP are both rate-limiting enzymes involved in the gluconeogenic and glycogenolytic pathways [18, 34]. The real-time PCR analysis indicated that TSFP could decrease the expression levels of GP and G6Pase, which could result in enhanced glycolysis and diminished gluconeogenesis [35]. Besides, the enhancement of glucose metabolism could be also possibly linked with the antioxidative effect of TSFP in vivo, which could be explained by the inhibition of lipid peroxidative injury and the enhancement of oxygen-carrying capacity after TSFP administration [16, 26].

The pathological findings regarding the organs related to diabetes indicated the presence of immunopathological inflammatory processes. It uncovered the damage to the organs caused by the immune system following the occurrence of the disease. Hence, in this study, the mRNA expression levels of the immune factors including IFN-γ, IL-4, T-bet, and GATA-3 were investigated [36, 37]. Naive T helper (Th) lymphocytes differentiate into two subsets, Type 1 (Th1) and Type 2 (Th2) effector cells [38, 39]. The cytokine produced by the Th1 subset is IFN-γ, and the signature cytokine of the Th2 subset is IL-4, and this requires the action of two opposing transcription factors of T-bet and GATA-3 [40]. The expression of IFN-γ/IL-4 and T-bet/GATA-3 commonly represent the polarization tendency of Th cells involved various pathological reactions and inflammatory processes [41]. The results indicated that the experimental group administered with TSFP could partially restrain excessive mRNA expression of cytokines and transcription factors. It could effectively reduce the Th1 polarization tendency in diabetic mice. This suggested that TSFP could effectively suppress the production of inflammatory cytokines and provide hepatic protection through translational regulation, signaling pathway activation, and mRNA processing [18, 42]. Besides, various amino acids contained in TSFP could also reduce inflammatory reactions in the livers of the diabetic mice [24]. Combined with the results of histopathological changes, it could be reasonably speculated that TSFP could promote tissue regeneration process, accelerate collagen synthesis, and improve anti-inflammatory cell proliferation for anti-inflammation enhancement and tissue damage alleviation.

The gut microbiota composition plays a key role in the health and well-being of mice, and the imbalances in gut microbiota may cause the progression of gut dysbiosis, pathogen infections, and metabolic diseases [18]. Bacterial alpha diversity is applied in analyzing the community richness and the complexity of species diversity [43]. An increase in diversity and richness can enhance the integrity and stability of the gut microbiota profile. Conversely, lower values may be associated with a weakened intestinal tract ecosystem [44]. In this study, the lowest indices of alpha diversity were found in the M group, indicating that the gut microbiome dysbiosis appeared in the diabetic mice, which would increase the susceptibility to pathogen invasion and make the digestive system perform more unsteadily [21]. Contrastively, the H group produced superior indices in terms of species richness and diversity, with the L group following close behind. This implied that TSFP would possess an ability to enrich specific members within the community, consequently enhancing the functional stability of the bacterial community and reducing the risk of diseases in the mice.

The gut microbiota composition of mice before administration of TSFP was similar to previous reports, and the observed bacteria were regarded as the indigenous microorganisms in the gut of mice [45]. This suggested that the gut microbiota profile maintained steady, and the animals were not infected. After administration of TSFP, the higher proportions of Bacteroidetes, Actinobacteria, and the lower proportions of Firmicutes and Proteobacteria were observed in the L and H groups. In general, Bacteroidetes were metabolically heterogeneous and capable of metabolizing carbohydrates, which could improve nutrient absorption and protein conversion [46]. The Actinobacteria was regarded as an essential microbial indicator related to biosynthesis and energy conversion [47]. Relatively, certain members of the Firmicutes phylum were correlated to metabolic diseases, and the Proteobacteria phylum had been proven to be a symbol indicative of intestinal microbiota disorders and intestinal inflammation [48].

At the genus level, increased abundances of potentially pathogenic bacteria related to organic damage and metabolic disorders, including *Clostridium*, *Enterococcus* and *Desulfobacterium*, were observed in the M group, which were present in very low abundances or even absent in the TSFP-treated groups. Previous report showed that diabetes could be accompanied by a high proportion of *Clostridium* spp [49]. The *Enterococcus* could increase the toxicity of the intestinal contents, and the *Desulfobacterium* could exert adverse effects by destroying intestinal mucosa, weakening intestinal barrier function and inducing inflammation [46, 50]. The abundances of *Bacteroides*, *Prevotella*, *Bifidobacterium* increased visibly in the TSFP-treated groups, which would contribute to a stable and versatile community structure. In some cases, *Bacteroidetes* could reduce inflammation by producing short chain fatty acids, enhancing the differentiation of goblet cells, and activating Bacteroides-folicacid-liver pathway during metabolism [51, 52]. *Bifidobacterium* could regulate cholesterol metabolism and convert cholesterol into steroids, further alleviating high blood pressure [21]. In addition, the *Bifidobacterium* would also play crucial roles in improving lipid metabolism disorders and inhibiting the growth of saprophytes [53]. *Prevotella* could produce acetic acid, iso-butyric acid and iso-valeric acid, which might prevent invading organisms and enhance immune response [54]. Besides, the emerging genera in the TSFP-treated groups, such as *Lactobacillus*, *Parabacteroides*, and *Lachnospira*, might produce potential benefits for hosts by providing energy source, improving normal metabolism, and exerting anti-inflammatory effects [55–57]. The results confirmed that the administration of TSFP could effectively shape and optimize the gut microbiota compositions of diabetic mice by enriching the beneficial bacteria and suppressing potentially pathogenic microorganisms.

The structure and function of gut microbial communities are related to the serum parameters of the mice [18]. To clarify the response relationships between them, the correlation analysis was performed in this study. In the M group, low correlations between the serum parameters and the abundances of functional bacteria such as *Lachnospira*, *Lactobacillus*, *Mucispirillum*, and *Parabacteroides* were observed. This indicated that the integrity and stability of the gut microbiota profile were diminished, and the risks of intestinal diseases were increased [58, 59]. In general, a greater number of species and a higher community evenness may indicate an abundant and diverse structure for the predominant species. On the other hand, a community with fewer species could be fragile and lack the ability to counteract disruptions from stress exposure and external invasions [44]. The abundances of pathogenic bacteria, including *Aerococcus*, *Clostridium*, *Desulfobacterium*, *Enterococcus*, and *Turicibacter*, showed significantly correlations with the serum parameters in the M group compared with the TSFP-treated groups. In fact, the metabolic disorders, intestinal inflammation, and digestive system disruption could be partially ascribed to the proliferations of these genera [45, 60]. The results could be explained that some specific taxonomic groups induced by ALX would become nutritional and spatial competitors to indigenous bacteria, and TSFP could reduce the abundances and weaken the functions of these microorganisms effectively. In addition, the abundances of functional bacteria and potential probiotics in the TSFP-treated groups exhibited better correlations with serum parameters than other groups, indicating that a stable and versatile gut microbiota profile had been established by TSFP treatment. As mentioned before, these bacteria appeared with different functions and roles in intestinal system, such as antioxidation, active acid formation, mucosal restitution, epithelial energy collection, cholesterol metabolism modulation, and intestinal barrier function enhancement [44, 49]. All these would be of positive significance in terms of boosting antioxidation, regulating blood glucose, and alleviating inflammation.

## Conclusions

To conclude, the present study confirmed that TSFP supplementation could alleviate hyperglycaemia effectively by enhancing antioxidative activities and reducing inflammatory damages of diabetic mice. In addition, TSFP supplementation showed a greater ability to shape and optimize the gut microbial composition of diabetic mice by enriching the beneficial bacteria and suppressing the pathogenic microorganisms, as well as exhibiting better correlation between the enrichment of functional bacteria and potential probiotics with serum parameters. From above all, supplementation of TSFP would serve as an effective and safe treatment approach for blood glucose regulation and inflammation remission, thus opening up a new perspective for the development of functional food additives to enhance anti-diabetic effects.

## Supporting information

S1 Graphical abstract (DOCX)
