# Supplementary material for: Anti-oxidation enhancement, inflammation alleviation, and microbial composition optimization of using tussah (Antheraea pernyi) silk fibroin peptides for hyperglycaemia remission
Source: PLoS One. 2025 Jan 23;20(1):e0317891. doi: 10.1371/journal.pone.0317891 (PMC11756761; doi:10.1371/journal.pone.0317891)
Supplement: Supplementary file 1 [file pone.0317891.s001.docx]

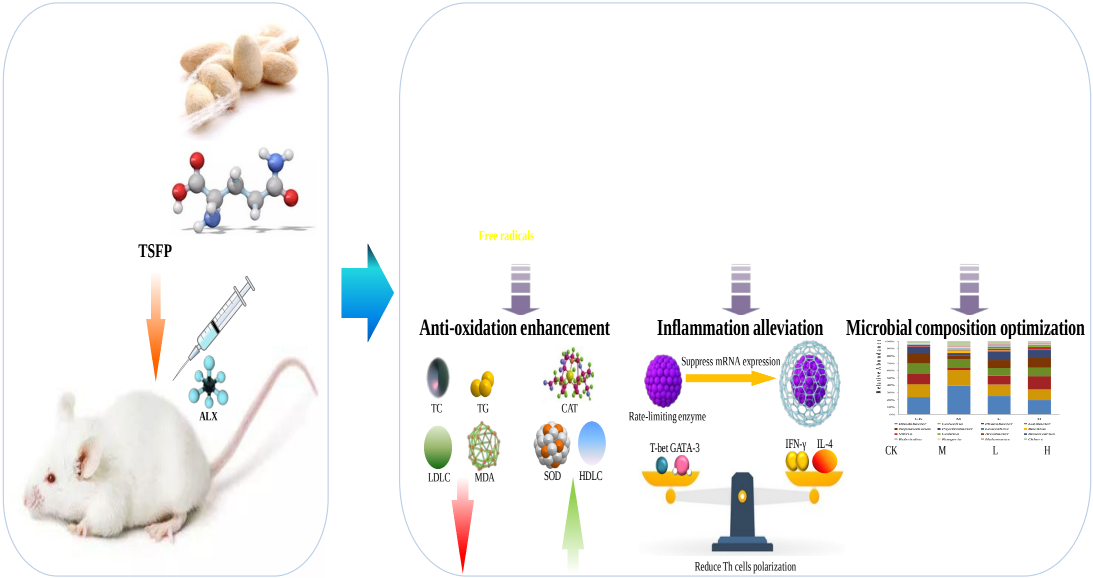
TSFP
ALX
Free radicals
Anti-oxidation enhancement
TC
TG
CAT
LDLC
MDA
SOD
HDLC
Inflammation alleviation
Suppress mRNA expression
Rate-limiting enzyme
T-bet GATA-3
IFN-γ
IL-4
Reduce Th cells polarization
Microbial composition optimization
CK
M
L
H
